# Generation of integration-free induced hepatocyte-like cells from mouse fibroblasts

**DOI:** 10.1038/srep15706

**Published:** 2015-10-27

**Authors:** Jonghun Kim, Kee-Pyo Kim, Kyung Tae Lim, Seung Chan Lee, Juyong Yoon, Guangqi Song, Seon In Hwang, Hans R. Schöler, Tobias Cantz, Dong Wook Han

**Affiliations:** 1 Department of Stem Cell Biology, School of Medicine, Konkuk University, 1 Hwayang-dong, Gwangjin-gu, Seoul 143-701, Republic of Korea; 2 Department of Cell and Developmental Biology, Max Planck Institute for Molecular Biomedicine, Röntgenstrasse 20, 48149 Münster, Germany; 3 REBIRTH Cluster of Excellence, Hannover Medical School, Hannover 30625, Germany; 4 University of Münster, Medical Faculty, Domagkstrasse 3, 48149 Münster, Germany; 5 KU Open-Innovation Center, Institute of Biomedical Science & Technology, Konkuk University, 1 Hwayang-dong, Gwangjin-gu, Seoul 143-701, Republic of Korea

## Abstract

The ability to generate integration-free induced hepatocyte-like cells (iHeps) from somatic fibroblasts has the potential to advance their clinical application. Here, we have generated integration-free, functional, and expandable iHeps from mouse somatic fibroblasts. To elicit this direct conversion, we took advantage of an oriP/EBNA1-based episomal system to deliver a set of transcription factors, *Gata4, Hnf1a*, and *Foxa3*, to the fibroblasts. The established iHeps exhibit similar morphology, marker expression, and functional properties to primary hepatocytes. Furthermore, integration-free iHeps prolong the survival of fumarylacetoacetate-hydrolase-deficient (Fah^−/−^) mice after cell transplantation. Our study provides a novel concept for generating functional and expandable iHeps using a non-viral, non-integrating, plasmid-based system that could facilitate their pharmaceutical and biomedical application.

Introducing lineage-specific transcription factors (TFs) into somatic cells enables the induction of distinct cellular identities without the need to first pass through a pluripotent stem cell (PSC) state^1^,^2^,^3^,^4^,^5^,^6^,^7^,^8^,^9^,^10^,^11^,^12^,^13^. We and others have demonstrated the direct conversion of somatic cells into adult stem cells or progenitor cells, such as angioblast-like progenitor cells, hematopoietic stem cells, and neural stem cells^14^,^15^,^16^,^17^,^18^,^19^,^20^,^21^,^22^,^23^,^24^,^25^,^26^,^27^,^28^. The process underlying direct conversion is known to be relatively simpler and faster than that of induced pluripotent stem cell (iPSC) generation. Furthermore, directly converted cells have been shown to exhibit therapeutic potential following transplantation into respective disease models without obvious evidence for tumor formation^29^,^30^. Thus, TF-mediated direct conversion technology has been considered as an alternative to iPSC technology for patient-specific cell- and tissue-replacement therapies.

Recent progress in the direct conversion field has also enabled the generation of hepatocyte-like cells, namely induced hepatocytes (iHeps), from mouse and human fibroblasts by the forced expression of different TF combinations^9^,^10^,^11^,^12^,^13^. Specifically, Huang *et al.* (2011) reported that ectopic expression of *Gata4, Hnf1a*, and *Foxa3* could induce the direct conversion of mouse fibroblasts into functional iHeps. Sekiya *et al.* (2011) identified three different combinations of TFs (*Hnf4a*/*Foxa1*, *Hnf4a*/*Foxa2*, and *Hnf4a*/*Foxa3*) whose overexpression in mouse fibroblasts enables the generation of functional iHeps. Furthermore, recent studies have shown that human iHeps (hiHeps) can be generated from fibroblasts using a similar strategy to mouse studies^11^,^12^,^13^. However, all the aforementioned studies have employed either a lenti- or retroviral transduction method for delivering TFs to the starting cells^9^,^10^,^11^,^12^,^13^. This method raises concerns about the risk for insertional mutagenesis and integration-associated genotoxicity, as viral vectors can randomly integrate into the host genome^31^,^32^,^33^,^34^. Furthermore, the continued expression and unexpected reactivation of exogenous TFs in iHeps are particularly problematic events, considering the clinical application of iHep technology, because the uncontrolled reactivation of the transgenes may affect the cellular functionality of iHeps. These obstacles have been extensively addressed for iPSCs, even though iPSC lines can be indefinitely expanded on a clonal level, allowing the identification of karyotypically unaltered iPSC clones that would not carry virally integrated transgenes at potentially harmful integration sites. These assays would not be applicable to iHeps or other directly converted cell types, as these cells were generated from a bulk population of starting cells and are not prone to clonal propagation, even if they have a high capacity for proliferation. Therefore, a dire need exists to generate integration-free iHeps using non-viral, integration-free methods. Several methods have been reported from earlier reprogramming studies, including synthetic mRNA, sendai virus, recombinant proteins, and episomal plasmids^35^,^36^,^37^,^38^,^39^,^40^,^41^,^42^. Although these methods have unique advantages, they also have disadvantages, such as high variability in their reprogramming efficiency^43^. We selected the oriP/Epstein-Barr Nuclear Antigen-1 (EBNA-1)-based episomal plasmid for delivering TFs to somatic cells, as it represents a technically simple, convenient, fast, and highly reproducible approach for generating integration-free cells^39^,^40^,^41^,^42^.

Here we show the generation of functional iHeps from both mouse embryonic and adult fibroblasts using episomal vectors containing *Gata4, Hnf1a*, and *Foxa3*. The generated iHeps are stably expandable *in vitro* and resemble primary hepatocytes in their morphology, gene expression pattern, and both *in vitro* and *in vivo* functionality. Notably, we were unable to detect the integration of exogenous TFs in iHeps, indicating that these cells are indeed free of transgenes. The novel approach defined in this study offers a method for obtaining a sufficient amount of clinically relevant hepatocyte-like cells that could expedite the clinical translation of iHeps.

## Results

### Generation of iHeps from embryonic fibroblasts using episomal vectors

**Figure 1:**
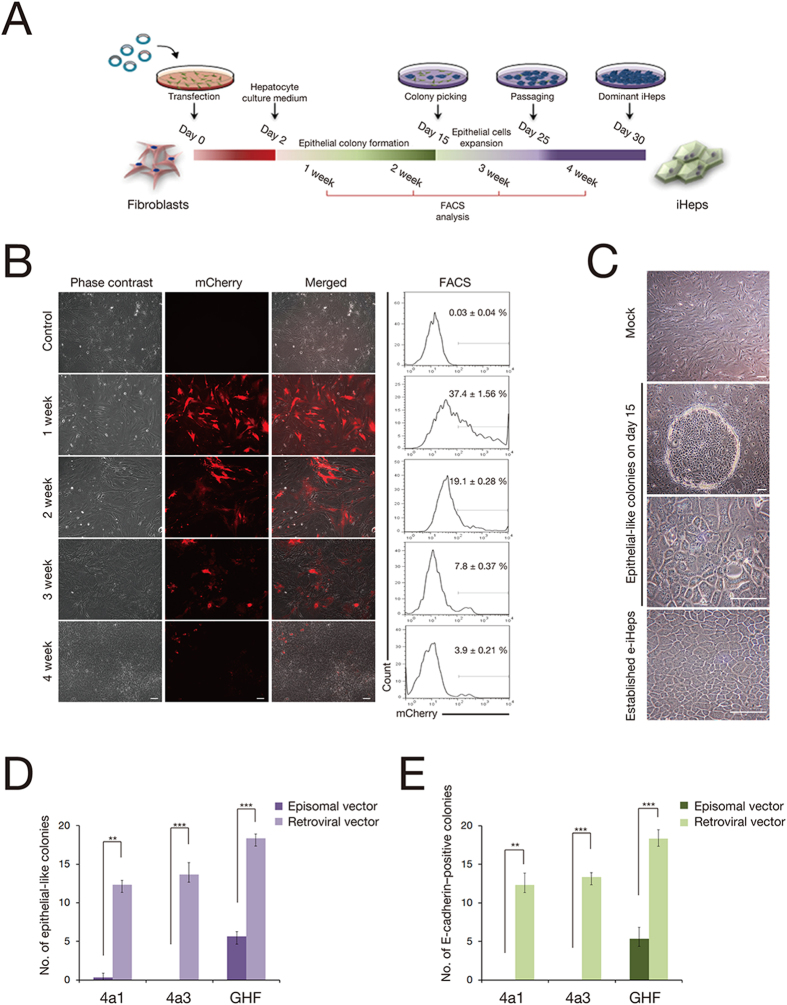
Generation of integration-free iHeps from fibroblasts by episomal vectors. (**A**) Schematic illustration depicting the procedure for the generation of integration-free iHeps. (**B**) Expression of mCherry after transfection was monitored by FACS analysis in a time-course manner. (**C**) The morphology of epithelial colonies on day 15 after transfection and established e-iHeps, as assessed by bright-field microscopy. Scale bars, 100 μm. (**D,E**) Number of epithelial colonies (**D**) and E-cadherin–positive colonies (**E**) on day 15 after transfection with different combinations of transcription factors. Error bars indicate the standard deviation of triplicate values. **P < 0.01, ***P < 0.001.

We first derived mouse embryonic fibroblasts (MEFs) from embryonic day 13.5 (E13.5) C57/B6 mouse embryos. MEFs were free of epithelial and hepatic tissues, as demonstrated by the lack of *Afp*, *Alb*, and *E-cadherin* gene expression (Supplementary Fig. S3C). As a quality control of an episomal expression system, we transfected MEFs with an episomal vector encoding mCherry by nucleofection and monitored mCherry expression over a period of 4 weeks (Fig. 1A,B). mCherry was expressed in 37.4 ± 1.56% cells at 1 week post-transfection. The number of mCherry-positive cells was dramatically reduced thereafter, and mCherry expression was detected in only 3.9 ± 0.21% cells at 4 weeks post-transfection. We also transfected MEFs with each episomal vector containing an individual TF (*Hnf4a, Foxa1, Foxa3, Gata4*, and *Hnf1a*) and monitored both the percentage of transfected cells and the expression level of each TF (Supplementary Fig. S1A and B). Quantitative polymerase chain reaction (qPCR) analyses from the transfected MEFs indicated that the TFs were efficiently expressed (Supplementary Fig. S1A). Notably, TF gene expression in MEFs was continuously down-regulated over subsequent passaging and barely detectable at passage 10. Immunofluorescence analyses on day 5 post-transfection indicated the detection of an individual TF in approximately 30% of MEFs (*Gata4*: 30.37 ± 5.64; *Hnf1a*: 33.23 ± 3.55; *Foxa3*: 32.21 ± 4.53; *Hnf4a*: 32.90 ± 5.91; and *Foxa1*: 30.64 ± 5.19; Supplementary Fig. S1B). The number of cells expressing the individual TF was continuously reduced upon serial passaging, and only a very few cells stained positive at 4 weeks post-transfection (*Gata4*: 1.10 ± 0.50; *Hnf1a*: 2.39 ± 1.53; *Foxa3*: 0.44 ± 0.88; *Hnf4a*: 2.11 ± 1.59; and *Foxa1*: 1.51 ± 1.49; Supplementary Fig. S1B).

We next transfected the MEFs with three distinct combinations of TFs, *Hnf4a*/*Foxa1* (4a1), *Hnf4a*/*Foxa3* (4a3), and *Gata4*/*Hnf1a*/*Foxa3* (GHF), which were previously shown to elicit the direct conversion of somatic fibroblasts into iHeps (Fig. 1A)^9^,^10^. On day 2 post-transfection, we supplied hepatocyte culture medium (HCM), which is known to support the growth of hepatic cells. On day 15 post-transfection, cells transfected with GHF displayed a homogeneous population of colonies that grew rapidly and exhibited typical epithelial morphology in culture (Fig. 1C). However, cells transfected with episomal vectors containing either 4a1 or 4a3 showed no or very few epithelial-like colonies in culture, indicating that episomal vector–mediated expression of these two combinations was rather insufficient for inducing direct conversion in this setting (Fig. 1D). Neither MEFs transfected with a vector containing mCherry alone nor untransfected MEFs cultured under identical culture conditions for the entire period yielded any epithelial-like colonies (Fig. 1C).

As GHF-transfected MEFs gave rise to distinct epithelial-like colonies, we attempted to assess the efficiency of this conversion process. We first counted the number of epithelial-like colonies on the culture plate. We identified 5.67 ± 0.58 colonies from three independent reprogramming experiments (Fig. 1D). To accurately measure the number of putative hepatocyte-like colonies, we fixed the cells and performed immunofluorescence with an antibody directed against E-cadherin. All the colonies arising from the GHF-transfected MEFs stained positive for E-cadherin (Supplementary Fig. S2A), whereas no E-cadherin–positive colonies were found in MEFs transfected with 4a1, 4a3, or mCherry (Fig. 1E). Untransfected MEFs and primary hepatocytes, used as controls, stained negative and positive for E-cadherin, respectively (data not shown). Of 1.5 × 10^5^ MEFs transfected, approximately 3.25% of cells were found to receive all three transgenes, *Gata4, Hnf1a*, and *Foxa3* (0.3037 × 0.3323 × 0.3221 = 0.0325 = 3.25%; Supplementary Fig. S1B). Thus, we conclude that of the 1.5 × 10^5^ starting cells, ~4,875 cells should carry all three episomal vectors. As 5.67 colonies were found to stain positive for E-cadherin, the conversion efficiency is estimated to be 0.12% (5.67/4875 = 0.00116 = 0.12%). In order to further confirm the generation of iHeps from MEFs transfected with distinct combinations, we next performed FACS analysis using more specific hepatic markers such as Albumin (ALB) and Alpha-1 antitrypsin (AAT). To this end, we stained the transfected MEFs from different reprogramming conditions for ALB and AAT on day 15 after gene delivery. In the line with E-cadherin staining data (Fig. 1E), we were able to observe both ALB- and AAT-positive cells with GHF but not with either 4a1 or 4a3 (Supplementary Fig. S2B). These data indicate that the single transfection of both 4a1 and 4a3 is not sufficient for generating iHeps, although the retroviral transduction of either 4a1 or 4a3 could readily generate iHeps.

A quality control of the TF-mediated direct conversion process, we generated iHeps in parallel using a retroviral system. We transduced MEFs with gamma-retroviruses containing 4a1, 4a3, and GHF, and cultured the cells under HCM for 15 days. Consistent with previous studies^9^,^10^, we readily obtained epithelial-like colonies (4a1: 12.33 ± 1.52; 4a3: 13.33 ± 0.58; GHF: 18.33 ± 1.15), which were also positive for E-cadherin (Supplementary Fig. S2A). We designated the cells as retrovirus-mediated iHeps (r-iHeps). Their conversion efficiency was found to be significantly higher than that of iHeps generated by an episomal system. The r-iHeps were stably expandable *in vitro* and exhibited a similar morphology and gene expression pattern to primary hepatocytes (see below).

### Characterization of episomal vector–mediated iHeps

**Figure 2:**
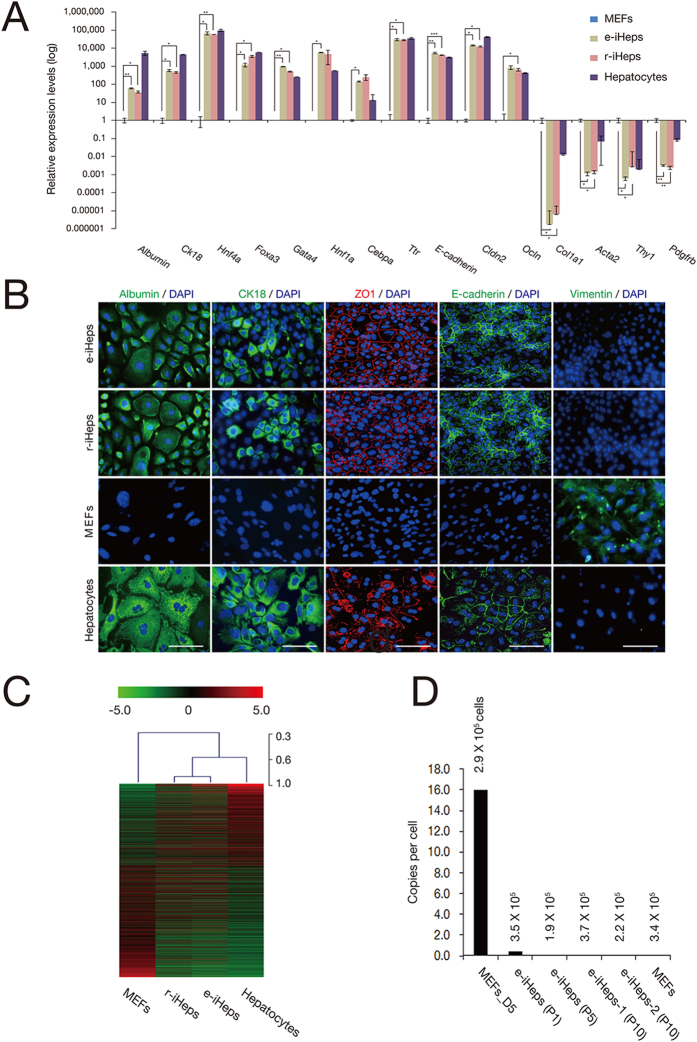
Characterization of e-iHeps. (**A**) Expression of hepatocyte and fibroblast markers was analyzed by qPCR in e-iHeps. Expression levels were normalized to those of MEFs. Error bars indicate the standard deviation of triplicate values. *P < 0.05, **P < 0.01, ***P < 0.001. (**B**) Immunofluorescence microscopy images of iHep lines using antibodies directed against albumin, CK18, and vimentin, and confocal microscopy images using antibodies directed against E-cadherin and ZO-1. MEFs and primary hepatocytes were used as negative and positive controls, respectively. Scale bars, 100 μm. (**C**) Heat map representing the global gene expression profile of MEFs (n = 1), e-iHeps (passage 10; n = 2), r-iHeps (passage 10; n = 2), and primary hepatocytes cultured for 6 days (n = 3). Genes that are more than 2-fold differentially expressed between MEFs and primary hepatocytes are represented. The color bar at the top indicates gene expression in log_2_ scale. Red and green colors represent higher and lower gene expression levels, respectively. Hierarchical clustering of the cell lines based on the gene expression profiles from the heat map is shown at the top of the heat map. (**D**) The number of copies of the episomal vectors in e-iHeps was analyzed by qPCR. Numbers indicate the estimated cell numbers examined in each cell line. MEFs on day 5 after transfection were used as a positive control.

We asked whether the E-cadherin–positive epithelial-like colonies generated by an episomal system possessed morphological, molecular, and functional properties typical of primary hepatocytes. To address this question, we manually picked colonies and enzymatically dissociated them, and then replated the individual cells and cultured them for 30 days. Their morphology was similar to those of r-iHeps and primary hepatocytes, but clearly distinct from that of the starting population, MEFs (Fig. 1C). The cells actively proliferated over 10 passages without losing their normal karyotype, whereas untransfected MEFs underwent cellular senescence (Supplementary Fig. S3A and B). RT-PCR and qPCR analyses indicated the activation of genes typical of hepatocytes (*Afp*, *Alb*, endogenous *Hnf1a*, endogenous *Foxa3*, endogenous *Gata4, Cebpa, Hnf4a*, *Ttr*, *Ck8*, *Ck18*, *Cldn2*, and *E-cadherin*) in E-cadherin–positive epithelial-like cells, indicating the establishment of an endogenous hepatic program. In contrast, genes specific to fibroblasts, such as *Acta2*, *Col1a1*, *Pdgfrb*, *Postn*, and *Thy1*, were efficiently down-regulated in the cells, indicating the loss of fibroblast-specific characteristics (Fig. 2A and Supplementary Fig. S3C). As the E-cadherin–positive epithelial-like cells closely resemble primary hepatocytes and r-iHeps, we designated these cells as episomal vector–mediated iHeps (e-iHeps). Of note, as the expression of both other primitive endodermal markers and hepatic stem cell markers was not detected in e-iHeps (Supplementary Fig. S3D), we conclude that the cells were directly converted from MEFs without first passing through either an endodermal progenitor cell state or a hepatic stem cell state.

We then confirmed activation of the endogenous hepatic program and inactivation of the fibroblast program at the protein level. Immunofluorescence analyses showed that hepatic markers including albumin, CK18, ZO-1, and E-cadherin were distinctly expressed in more than 80% of the e-iHeps. These markers were comparably expressed in primary hepatocytes and r-iHeps, but were not expressed in the starting cells, MEFs. The fibroblast maker, vimentin, was expressed in unconverted MEFs—but not in e-iHeps, r-iHeps, and primary hepatocytes (Fig. 2B).

We next analyzed the global gene expression profile of the different cell lines by microarray (Fig. 2C). Heat map analysis indicated that e-iHeps closely clustered together with r-iHeps and primary hepatocytes, but were clearly separated from MEFs. Particularly, genes known to be involved in hepatic metabolisms and related functions, such as *Apoa1, Cyp39a1, Crot*, and *Akr1c13*, were up-regulated in both e-iHeps and r-iHeps, compared to MEFs. Conversely, genes typically expressed in fibroblasts, such as *Twist1*, *Snai2*, and *Zeb2*, were significantly down-regulated in both e-iHeps and r-iHeps, compared to MEFs (Supplementary Fig. S3E). Notably, although iHeps and primary hepatocytes exhibited a similar gene expression pattern, substantial differences between the two lines were also observed, consistent with the results of previous studies^9^,^10^.

We next estimated the number of copies of the episomal vectors per cell with progression of conversion and with serial passaging (Fig. 2D). We detected 16 copies of the vectors per cell on day 5 after transfection, but the copy numbers were significantly decreased at passage 1 (0.46 copies). Of the 2 e-iHep clones tested at passage 10 (40 days after transfection), both contained only less than 0.05 copies of the transgenes, indicating that e-iHeps do not show any evidence for transgene integration. Thus, we could eliminate the potential integration of exogenous TFs in the host genome using an episomal system.

### Assessing *in vitro* and *in vivo* functionality of e-iHeps

**Figure 3:**
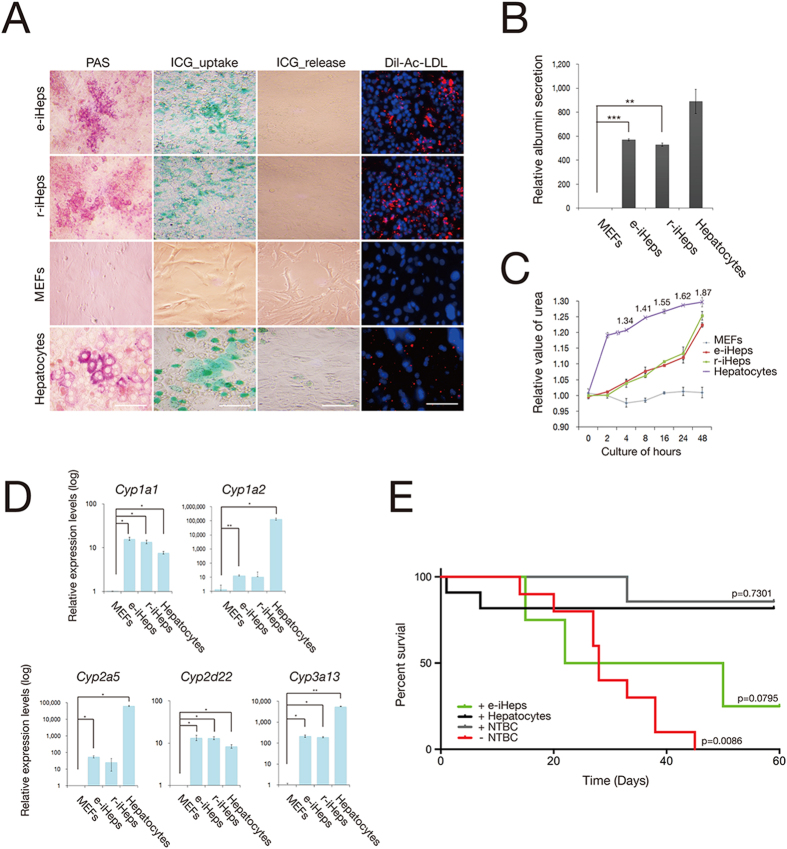
Functional analysis of e-iHeps. (**A**) *In vitro* functional analysis of e-iHeps including PAS staining, ICG uptake assay, and intake of acetylated low-density lipoprotein labeled with the fluorescent probe Dil-Ac-LDL. MEFs and primary hepatocytes were used as negative and positive controls, respectively. Scale bars, 100 μm. (**B,C**) Both albumin secretion (**B**) and urea production (**C**) were assessed in e-iHeps. MEFs and primary hepatocytes were used as negative and positive controls, respectively. Error bars indicate the standard deviation of triplicate values. **P < 0.01, ***P < 0.001. (**D**) Expression levels of CYP450 genes in e-iHeps after treatment with inducers (3-methylcholanthrene, rifampicin, and dexamethasone) by qPCR. Expression levels were normalized to those of MEFs. Error bars indicate the standard deviation of triplicate values. *P < 0.05, **P < 0.01. (**E**) Cumulative survival blot of FRG mice after transplantation of e-iHeps (n = 4) and primary hepatocytes (n = 10) in comparison with drug-treated ( + NTBC) and untreated (-NTBC) animals.

We next assessed the *in vitro* functionality of e-iHeps using multiple assays. Periodic Acid-Schiff (PAS) staining revealed glycogen storage in more than 70% of the e-iHeps. Indocyanine green (ICG) uptake analyses proved the xenobiotic metabolic activities in more than 50% of the e-iHeps. Furthermore, e-iHeps were found to transport DiI-Ac-LDL into the cytoplasm comparably to primary hepatocytes and r-iHeps (Fig. 3A). The e-iHeps secreted albumin and produced urea comparably to primary hepatocytes (Fig. 3B,C).

Cytochrome P450 (CYP450) enzymes play a key role in drug metabolisms in hepatocytes^44^. We first determined the expression levels of *Cyp1a1*, *Cyp1a2*, *Cyp2a5*, *Cyp2d22*, and *Cyp3a13* in e-iHeps after treatment with inducers (3-methylcholanthrene, rifampicin, and dexamethasone). Upon treatment with the inducers, e-iHeps exhibited significantly increased expression levels of all the CYP450 tested (Fig. 3D).

We next investigated the *in vivo* functionality of e-iHeps using a well-studied murine model of tyrosinemia type I, the fumarylacetoacetate-hydrolase-deficient (Fah^−/−^) mice^45^,^46^. We transplanted 1 × 10^6^ e-iHeps or 1 × 10^6^ primary hepatocytes into the livers of immunodeficient Fah^−/−^–Rag2^−/−^–γc^−/−^ mice (FRG mice) via intrasplenic injection (Fig. 3E). As expected, primary hepatocyte transplantation led to robust long-term survival, and thus only a minor alteration of the survival rate was detected in comparison to NTBC-treatment. The p-value for this comparison was 0.7301. In contrast, NTBC-withdrawal resulted in subacute liver failure and all mice died within 45 days (p < 0.01). The transplantation of e-iHeps results in a short-term liver support with reduced liver failure and ameliorated survival data. Consequently, we could observe survival beyond day 45 and the data set for e-iHep transplantation yielded in a p-value of 0.0795. These data suggest that e-iHeps harbor a considerable potential to support liver function, which is less powerful than primary hepatocyte transplantation.

### Generation of e-iHeps from adult fibroblasts using episomal vectors

**Figure 4:**
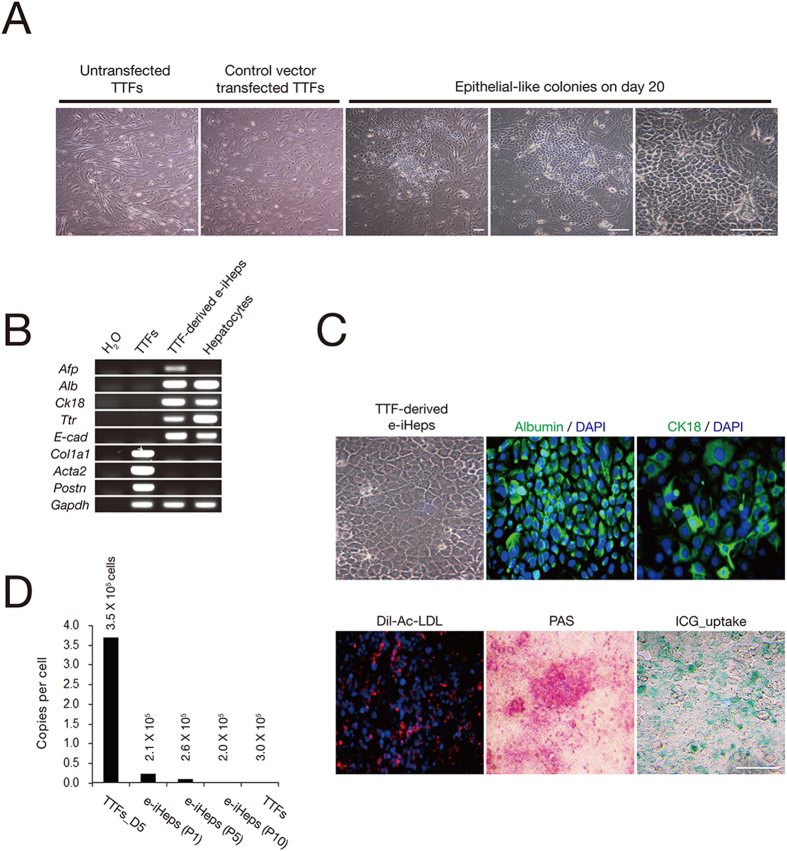
Generation of e-iHeps from adult fibroblasts. (**A**) The morphology of epithelial colonies from TTFs on day 20 after transfection. Scale bars, 100 μm. (**B**) RT-PCR analysis of TTF-derived e-iHeps. (**C**) The morphology of established TTF-derived e-iHeps; immunofluorescence microscopy images of TTF-derived e-iHeps using antibodies directed against albumin and CK18. *In vitro* functionality of TTF-derived e-iHeps was also determined by PAS staining, ICG uptake assay, and intake of acetylated low-density lipoprotein labeled with the fluorescent probe Dil-Ac-LDL. Scale bars, 100 μm. (**D**) The number of copies of the episomal vectors in TTF-derived e-iHeps. Numbers indicate the estimated cell numbers examined in each cell line. TTFs on day 5 after transfection were used as a positive control.

We asked whether the episomal system would also enable the generation of e-iHeps from adult fibroblasts. To address this question, we derived tail-tip-fibroblasts (TTFs) from 6-week-old mice and transfected them with episomal vectors containing GHF. The transfected cells were cultured for 20 days in HCM. We observed 3 E-cadherin–positive epithelial-like colonies in culture. The conversion efficiency of the adult fibroblasts was seemingly much lower than that of MEFs. Nevertheless, we were able to establish stably expandable TTF-derived e-iHeps (Fig. 4A). All hepatic maker genes examined were found to be readily expressed in TTF-derived e-iHeps, whereas genes expressed specifically in fibroblasts were efficiently down-regulated (Fig. 4B). Furthermore, albumin and CK18 were expressed in more than 70% of TTF-derived e-iHeps. These cells were found to transport DiI-Ac-LDL into the cytoplasm. PAS staining revealed glycogen stores in more than 50% of e-iHeps. Indocyanine green (ICG) uptake analyses revealed xenobiotic metabolic activities in more than 40% of e-iHeps (Fig. 4C). Finally, the e-iHeps were shown to be free from integration of viral transgenes (Fig. 4D).

## Discussion

iHeps could be a valuable tool for the *in vitro* modeling of liver diseases to investigate the underlying pathophysiology in an authentic cellular model. Furthermore, iHeps generated from patient specimens and pharmaceutically active small molecules can be a novel platform for testing patient-specific drug toxicity. Finally, they can potentially serve as an unlimited source of autologous cells for replacing damaged liver tissues. Thus, generating large quantities of iHeps could facilitate cell-based therapies, pharmaceutical applications, and *in vitro* disease modeling. In the current study, we attempted to generate functional and expandable iHeps using a non-viral, non-integrating, and plasmid-based system. We cloned hepatic TFs into the oriP/EBNA1-based episomal vectors. After delivering the vectors into MEFs by nucleofection, we obtained typical hepatocyte-like colonies, namely episomal vector–mediated iHeps (e-iHeps). The e-iHeps exhibit typical hepatocyte features in numerous aspects. Firstly, endogenous hepatic programs are fairly activated in e-iHeps, whereas fibroblast-specific gene expression becomes completely silenced. Secondly, e-iHeps display functional features typical of mature hepatocytes, such as albumin secretion, glycogen storage, Dil-Ac-LDL intake, and ICG uptake. Thirdly, upon treatment with Cyp inducers, e-iHeps express CYP450 enzymes. Furthermore, engrafted e-iHeps can substantially prolong the survival of FRG mice without NTBC. Finally, all e-iHep lines generated in the current study are integration free. Furthermore, in contrast to previous study^47^, our preliminary data from a follow-up study demonstrates that only three out of 12 analyzed e-iHep lines depict aberrant expression of the intestine marker gene Cdx2 (data not shown).

Transgene integration into the host genome by a viral transduction system could potentially cause insertional mutations and integration-associated genotoxicity^31^,^32^,^33^,^34^. Furthermore, it can cause uncontrolled reactivation of transgenes in the host cells. A recent study has described that integrated transgenes are anomalously reactivated during the differentiation of iPSCs. Consequently, the cells are unable to differentiate into the desired cell type^48^,^49^. Thus, the virus-mediated delivery system is associated with risks and should essentially be avoided when considering reprogramming/converting cells and using the resulting cells for clinical applications.

We found that the conversion efficiency of fibroblasts into hepatocyte-like cells using an episomal system is about 3-fold lower (e-iHeps: 0.12%) than that using a retrovirus-mediated delivery system (r-iHeps: 0.37%). This can be explained partly by the low transfection efficiency. In the current study, about 30% of cells were transfected with an individual TF by nucleofection (Supplementary Fig. S1B). In contrast, the retroviral transduction system allows for approximately 60% of MEFs to be infected with single TF (Supplementary Fig. S1C). One could argue that the higher initial transfection efficacy correlates with a higher average transgene copy number in triple-transfected cells, a notion that would support the direct conversion process. Moreover, with continued cell proliferation, there would be progressive dilution of the episomal expression of TFs, resulting in the gradually decreased expression of lineage-converting TFs—and thus a reduction in the direct conversion efficiency.

The generation of integration-free iHeps from fibroblasts using synthetic modified mRNA has recently been reported^50^. However, this method is tedious, and requiring repetitive transfections for a period of 2 weeks. Furthermore, the preparation of mRNA entails multiple steps, and is time-consuming and technically challenging. In contrast, our method is very simple, fast, convenient, and highly reproducible. First, a single transfection is sufficient to yield iHeps within 15 days. Second, the preparation of episomal plasmids is simple and routine, and can be done within 2 days. Third, the resulting plasmids are very stable *in vitro—*without extra care. Finally, the transfection protocol is well optimized and takes only 30 minutes in total. Thus, our method is practically and technically easier than the synthetic modified mRNA method.

At this stage the characteristics of iHeps as authentic liver metabolism model or functional cell transplant are controversially discussed, and clearly further work needs to be performed to identify additional supportive factors such as additional transcription factors, small molecules, and modulating microRNAs. In a recent study that evaluated cell-based therapies for acute and chronic liver failure, survival data of Fah^−/−^ mice was compared after transplantation of primary hepatocytes, fetal liver cells, iHepSCs, and bone marrow stem cells^51^. Our findings support this study with the notion that induced hepatic cells have a considerable effect on the survival of Fah-deficient mice, but are not functionally equivalent to primary hepatocytes in such an transplantation assay.

In summary, we describe the generation of integration-free iHeps from mouse fibroblasts using the oriP/Epstein-Barr Nuclear Antigen-1 (EBNA-1)-based episomal plasmid. To the best of our knowledge, this is the easiest and fastest method for generating integration-free iHeps. Further optimization of the transfection methodology and culture conditions would probably lead to the generation of integration-free human iHeps. Our novel approach may thus bring iHeps one step closer to finding clinical utility as cell-based therapy.

## Methods

### Ethics statement

All mice used were bred and housed at the mouse facility of Konkuk University (KU) or Hannover Medical School. Animal handling was in accordance with the respective institutional animal protection guidelines. This study was approved by Institutional Animal Care and Use Committee (IACUC).

### Cell culture

Mouse embryonic fibroblasts (MEFs) were isolated from E13.5 wild-type C57/B6 mouse embryos after carefully removing the head and all the internal organs, including the liver. MEFs were maintained in DMEM (Biowest) containing 10% fetal bovine serum (FBS) (Biowest), 5 ml of MEM/NEAA (Gibco), and 5 ml of penicillin/streptomycin/glutamine (PSG) (Invitrogen). In all experiments, MEFs were not split more than three times to avoid cellular senescence.

Tail-tip fibroblasts (TTFs) were derived from the tail-tip of 6-week-old C57/B6 mice. Briefly, a 2 cm–long tail tips were washed with PBS (Biowest), the superficial dermis was peeled away, and the remaining tissue was cut into 1 mm pieces with a scalpel. After that, the pieces were plated onto gelatin-coated dishes and cultured in MEF medium. Cells migrating out from the pieces were transferred and expanded further.

Primary hepatocytes were isolated from the livers of 8- to 10-week-old C57/B6 mice by the traditional collagenase perfusion method. Hepatocytes and iHeps were cultured in hepatocyte culture medium (HCM), consisting of DMEM/F-12 (Invitrogen) supplemented with 10% FBS, 0.1 μM dexamethasone (Sigma), 10 mM nicotinamide (Sigma), 1% insulin-transferrin-selenium (ITS) premix (Gibco), 5 ml of PSG, 10 ng/ml fibroblast growth factor 4 (FGF4) (Peprotech), 10 ng/ml hepatocyte growth factor (HGF) (Peprotech), and 10 ng/ml epidermal growth factor (EGF) (Peprotech).

### Generation of iHeps

The coding regions of *Hnf4a*, *Foxa1*, *Foxa3*, *Gata4*, *Hnf1a*, and mCherry were amplified by PCR and cloned into pCR8/GW/TOPO (Invitrogen) to create donor plasmids. The inserts in the donor plasmids were transferred into the destination plasmids pCXLE-gw (37626) (Addgene) and pMX-gw (18656) (Addgene) by the LR reaction.

To generate e-iHeps, 1.5 × 10^5^ fibroblasts were transfected with 1.5 μg of each episomal vector using the Amaxa P4 Primary Cell 4D-Nucleofector kit (Lonza) according to the manufacturer’s instructions. The transfected cells using the program setting CZ-167 were plated onto collagen-coated dishes and cultured in MEF medium for 2 days. On day 2 post-transfection, HCM was supplied to the cells, and the medium was changed every other day. To generate r-iHeps, fibroblasts were seeded at the density of 2.5 × 10^4^ cells per well of 6-well plates and incubated with retrovirus-containing supernatants together with 8 μg/ml of protamine sulfate (Sigma) for 2 days. On day 2 post-transduction, the medium was replaced with HCM, and the medium was changed every other day.

### RNA Isolation, Reverse-Transcription PCR, and Quantitative PCR

Total RNA was isolated using the RNeasy Mini Kit (QIAGEN) according to the manufacturer’s instructions. 1 μg of total RNA was reverse transcribed into complementary DNA (cDNA) using the High Capacity cDNA Reverse Transcription Kit (Applied Biosystems) according to the manufacturer’s instructions. RT-PCR was performed using the GoTaq Green Master Mix (Promega). qPCR was performed using the SYBR Green PCR Master Mix (Applied Biosystems) on the ABI 7500 Real-Time PCR system (Applied Biosystems). Relative gene expression levels were calculated using the 2^−∆∆Ct^ method. All quantifications were normalized to an endogenous *Gapdh* control. The unpaired t-test was performed for statistical significance. Primer sequences are described in Supplementary Table S1.

### Karyotyping

Karyotyping was performed at the Korea Research of Animal Chromosomes (KRACH) using standard protocols for high-resolution G-banding. Briefly, iHeps cultured in 25 T flasks were treated with 10 μg/ml colcemid (Gibco) for 4 h to arrest cells in metaphase. Cells were then incubated with hypotonic solution for 25 min at 37 °C. Flasks were then shaken to suspend the cells. After centrifugation, cells were resuspended in fixative solution (methane : acetic acid = 3 : 1). Resuspended cells were transferred onto cold wet slides, and the slides were then subjected to trypsin and Giemsa (Sigma) for GTG-banding.

### Immunofluorescence and Flow cytometry

Cells were washed with PBS and fixed with 4% paraformaldehyde (Sigma) for 20 min at room temperature. The fixed cells were incubated with 0.3% Triton X-100 (Sigma) with 5% FBS in PBS for 2 h at room temperature. Cells were then incubated with the following primary antibodies overnight at 4 °C: mouse anti-albumin (R&D Systems), mouse anti-CK18 (Abcam), rabbit anti-ZO-1 (Invitrogen), rabbit anti-E-cadherin (Cell Signaling), mouse anti-vimentin (Abcam), mouse anti-Hnf4a (Abcam), goat anti-Hnf1a (Santa Cruz), rabbit anti-Foxa1 (Abcam), goat anti-Foxa3 (Santa Cruz), and rabbit anti-Gata4 (Millipore). After incubating with the primary antibody, the cells were washed three times with PBS and incubated with the appropriate fluorescently labeled Alexa-Fluor secondary antibody for 2 h at room temperature in the dark. Nuclei were counterstained with Hoechst33342 (Invitrogen).

Fluorescence-activated cell sorting (FACS) analysis was performed using a FACS Aria I (BD biosciences). Flow cytometry data were analyzed using the FlowJo software version 9.1 (Tree Star).

### Dil-Ac-LDL, PAS, and ICG assays

Cells were incubated with 10 μg/ml acetylated LDL labeled with 1,1′-dioctadecyl-3,3,3′,3′-tetramethylindo-carbocyanine perchlorate (Dil-Ac-LDL) (Invitrogen) for 4 h at 37 °C. Cell nuclei were counterstained with Hoechst33342. Images were acquired using a Zeiss Axiovert200 inverted fluorescence microscope equipped with an AxioCam HRm camera.

Periodic Acid-Schiff (PAS) staining was performed using the Periodic Acid-Schiff kit (Invitrogen) according to the manufacturer’s instructions. Briefly, cells were fixed with 10% formalin in 95% cold ethanol, rinsed in slowly running tap water for 1 min, and then exposed to periodic acid solution for 5 min at room temperature. After rinsing three times with distilled water, the cells were treated with Schiff’s reagent for 15 min at room temperature and washed three times with tap water for 5 min. Images were acquired using an Olympus CKX41 microscope with a Canon EOS 600D camera.

For the indocyanine green (ICG) uptake assay, the cells were incubated with ICG solution (Sigma) at 37 °C for 1 h and washed three times with PBS. Images were acquired using an Olympus CKX41 microscope with a Canon EOS 600D camera.

### Albumin and Urea secretion assays

For measuring the amounts of mouse albumin and urea in the culture media, the collected culture supernatants from different cell types (MEFs, iHeps, and primary hepatocytes) were analyzed using the Mouse Albumin ELISA Kit (Bethyl Laboratories) and QuantiChrom Urea Assay Kit (BioAssay Systems), respectively, according to the manufacturer’s instructions.

### Microarray

100 ng of total RNA per sample was used as input into a linear amplification protocol, which involved synthesis of T7-linked double-stranded cDNA and 16 h of *in vitro* transcription incorporating biotin-labeled nucleotides. Purified and labeled cRNA was then hybridized to Mouse Genome 430 PM Array Strip (Affymetrix) for 16 h according to the manufacturer’s instructions. The array chip was stained on the Affymetrix fluidics station, and intensities of hybridized probes were determined using the Affymetrix imaging station. Raw data were background corrected and subsequently normalized using Partek Express Affymetrix Edition under the Robust Microarray Analysis (RMA) algorithm.

Of 45,141 total annotated genes, 3,276 genes found to be differently expressed between MEFs and primary hepatocytes by more than two fold were selected for analyses. Heat maps and hierarchical clustering of samples were generated using the MeV software. In addition, genes related to cytochromes, MET, and various metabolic pathways, such as those for xenobiotic, fat, cholesterol, and glucose metabolism, were selected from the previous study^10^. Original data were uploaded to the Gene Expression Omnibus database (accession number GSE67362).

### Copy number detection

To determine the number of copies of the episomal vector per cell, a serially diluted vector and genomic DNA were used for generating a standard curve. The relationship between 2^−∆∆Ct^ values and copy numbers was determined by the Ct value of EBNA-1. The Ct value for *Fbxo15* was used for determining the relationship between 2^−∆∆Ct^ values and cell numbers. Primer sequences are described in Supplementary Table S1.

### Cell transplantation

1 × 10^6^ iHeps and primary hepatocytes were transplanted into FRG mice carrying a triple knockout of Fumarylacetoacetate-hydrolase, Rag2, and common gamma chain. The cells were delivered into the liver through the portal vein by intrasplenic injection, and NTBC (2-[2-nitro-4-(trifluoromethyl)benzoyl]cyclohexane-1,3-dione, also known as Nitisinone) was withdrawn from the drinking water one day after surgery. Non-transplanted mice were fed drinking water in the presence or absence of NTBC as controls. A survival curve was drawn as a step function by the Kaplan-Meier method. We compare our findings with previous data, namely the survival rate of non-transplanted Fah^−/−^ mice after NTBC withdrawal from the study by Huang *et al.*^9^.

### Statistical analysis

For the statistical evaluation of our data set, we employed the unpaired t-test throughout this study. All the values are from at least triplicated analysis. *P* values were presented as *P < 0.05; **P < 0.01; ***P < 0.001.

## Additional Information

**How to cite this article**: Kim, J. *et al.* Generation of integration-free inducedhepatocyte-like cells from mouse fibroblasts. *Sci. Rep.* **5**, 15706; doi: 10.1038/srep15706 (2015).

## Supplementary Material

Supplementary Information
