# Supplementary material for: Generation of integration-free induced hepatocyte-like cells from mouse fibroblasts
Source: Sci Rep. 2015 Oct 27;5:15706. doi: 10.1038/srep15706 (PMC4621602; doi:10.1038/srep15706)
Supplement: Supplementary Information [file srep15706-s1.docx]

Supplementary information for

**Generation of integration-free induced**

**hepatocyte-like Cells from mouse fibroblasts**

Jonghun Kim, Kee-Pyo Kim, Kyung Tae Lim, Seung Chan Lee, Juyong Yoon, Guangqi Song, Seon In Hwang, Hans R. Schöler, Tobias Cantz & Dong Wook Han


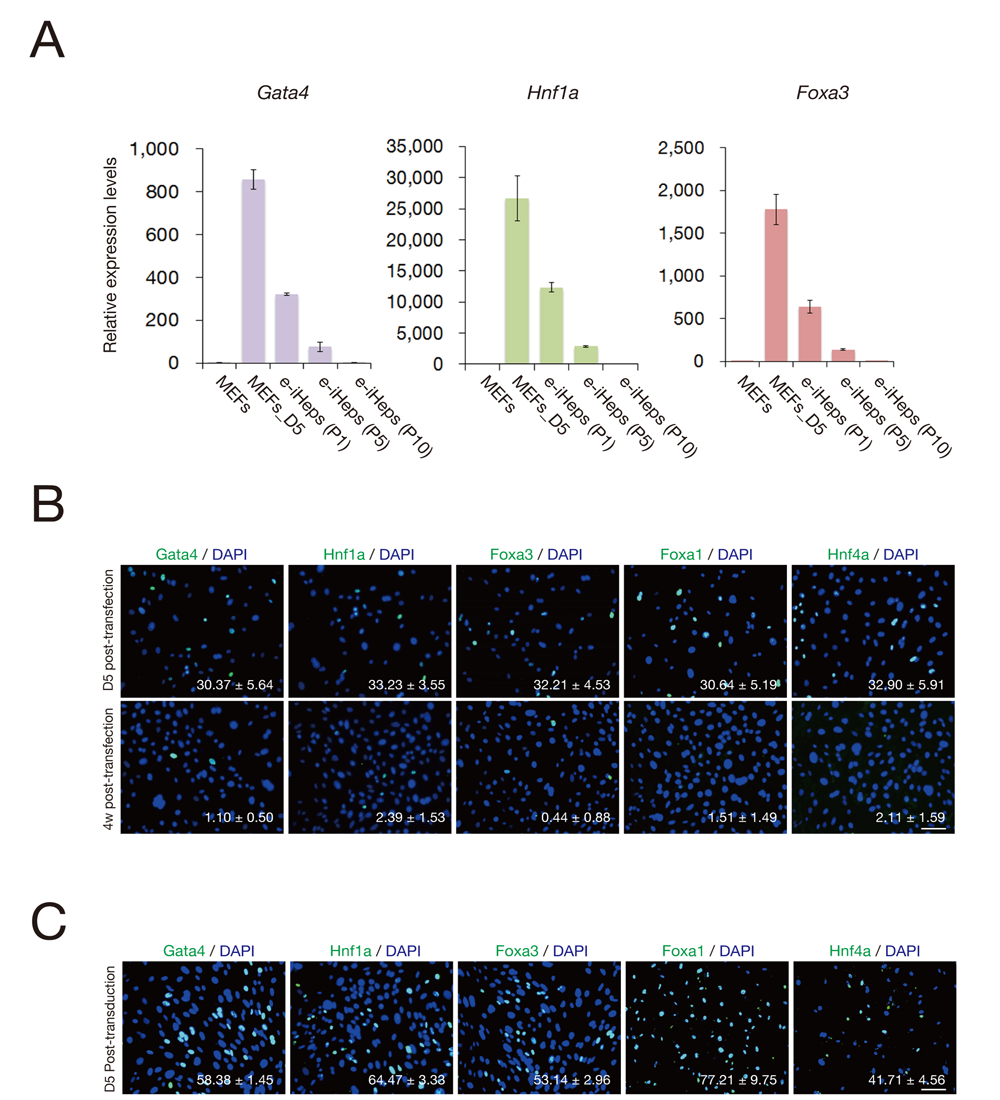


**Supplementary Figure S1. Transfection efficiency and induction levels of individual transgenes**. (A) Transgene expression levels were analyzed by qPCR and were normalized to those of MEFs. MEFs on day 5 after transfection were used as a positive control. (B) Transfection efficiency of individual transgenes was determined by immunostaining for the individual factors. The number of cells expressing the individual transgenes after 4 weeks of transfection was also determined by immunostaining. The values are from 4 independent transfection experiments. Scale bars, 100 µm. (C) Viral transduction efficiency of individual factors was assessed by immunostaining. Scale bars, 100 µm.


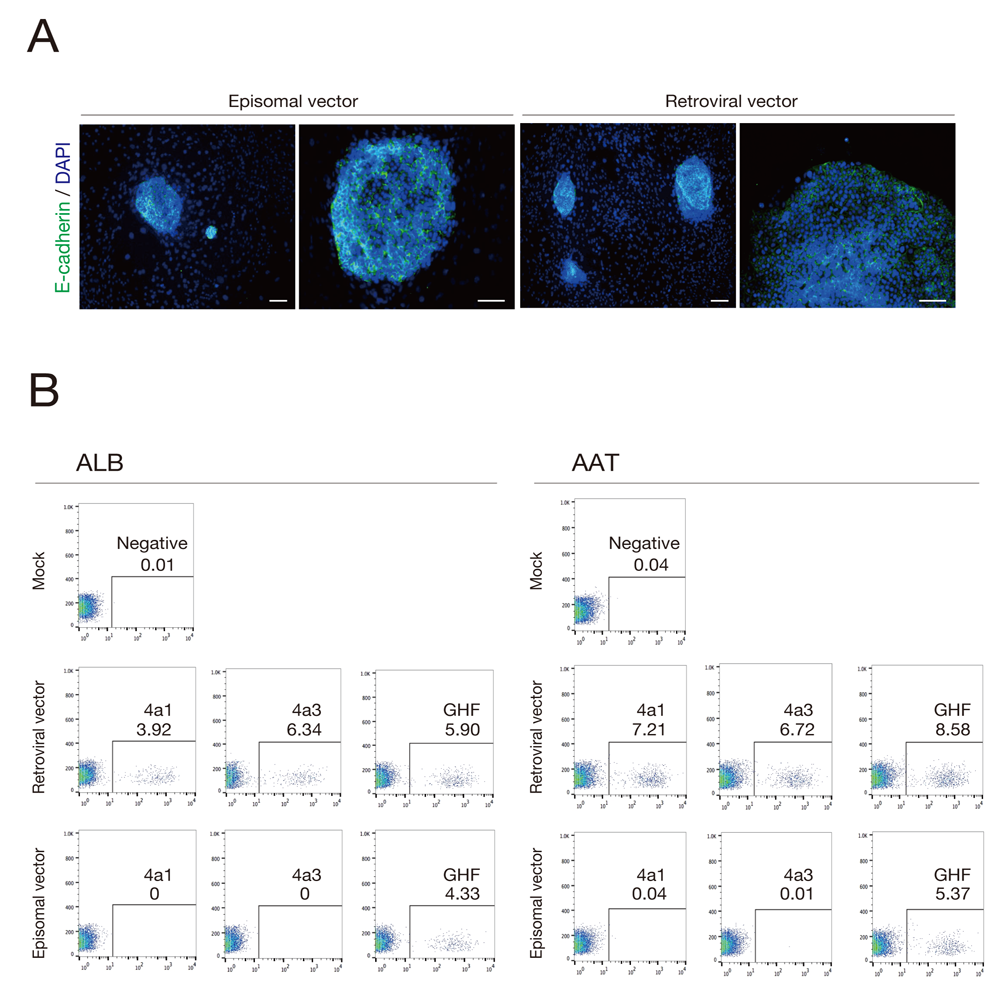


**Supplementary Figure S2. Efficiency of iHep generation from different reprogramming conditions.** (A) Immunostaining of E-cadherin–positive colonies on day 15 of transgene introduction by different gene delivery systems. Scale bars, 100 µm. (B) The efficiency of iHep generation was determined by FACS using antibody against ALB or AAT on day 15 after gene introduction.


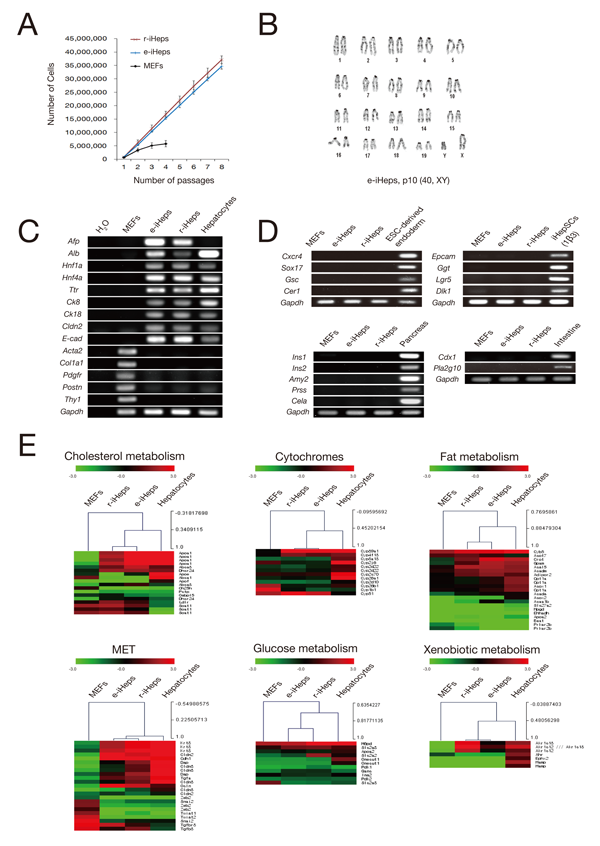


**Supplementary Figure S3. Characterization of e-iHeps. (**A) Proliferation rates of e-iHeps and r-iHeps. 1.5 x 10^5^ cells were passaged every 2 days on the wells of 12-well plates. Error bars indicate the standard deviation of triplicate values. (B) Karyotype analysis of e-iHeps at passage 10. (C) RT-PCR analysis of hepatocyte and fibroblast markers in iHep lines. *Gapdh* was used as a positive control. (D) Expression of endodermal progenitor, hepatic stem cell, pancreatic, and intestinal marker genes in both e-iHeps and r-iHeps was analyzed by RT-PCR. *Gapdh* was used as a positive control. (E) Heat map analysis showing the expression patterns of genes involved in different metabolic pathways in e-iHeps. Genes that are differentially expressed more than 2-fold between MEFs and primary hepatocytes are represented. The color bar at the top indicates gene expression in log_2_ scale. Red and green colors represent higher and lower gene expression levels, respectively. Hierarchical clustering of the cell lines based on the gene expression profiles from the heat map is shown at the top of the heat map.

**Supplementary Table S1. Primers used for RT-PCR and qPCR.**

| **Gene Name** | **Genebank Number** | **Primers** |
| --- | --- | --- |
| *Afp* | NM_007423 | 5’- CGTGATGCTTTGGGCGTTTA -3’ |
|  |  | 5’- GCCAAAAGGCTCACACCAAAG -3’ |
| *Alb* | NM_009654 | 5’- AAACCTTGTCACTAGATGCAAAGACG -3’ |
|  |  | 5’- GGGTAGCCTGAGAAGGTTGTGG -3’ |
| *Hnf1a* | NM_009327 | 5’- CCTGCTGCCATCCAACCATA -3’ |
|  |  | 5’- CCACGGTTACTGGGAAGAGGA -3’ |
| *Hnf4a* | NM_008261 | 5’- GCCAACGATCACCAAGCAAG -3’ |
|  |  | 5’- TGAGGGTATGAGCCAGCAGAA -3’ |
| *Ttr* | NM_013697 | 5’- CCCTGCTCAGCCCATACTCCTA -3’ |
|  |  | 5’- TGCTTTGGCAAGATCCTGGT -3’ |
| *CK8* | NM_031170 | 5’- AAGCTGGTGTCCGAGTCTTCTGA -3’ |
|  |  | 5’- AGCTCAGGCTGGCAAGGACT -3’ |
| *CK18* | NM_010664 | 5’- GATCGTGGATGGCAGAGTGG -3’ |
|  |  | 5’- TTCCCTCCTTCTCTGCCTCAGT -3’ |
| *Cldn2* | NM_016675 | 5’- TCTGCTCAACAGCCCAAAGC -3’ |
|  |  | 5’- TGGTTCTTCACACATACCCAGTCA -3’ |
| *E-cadherin* | NM_009864 | 5’- TTCAAGAAGCTGGCGGACAT -3’ |
|  |  | 5’- CATCTCCCATGGTGCCACAC -3’ |
| *Ocln* | NM_008756 | 5’- TCGCACATCAAGAGGATGGTG -3’ |
|  |  | 5’- GCCTCTGGAGAGAATTGCAGAGA -3’ |
| *Col1a1* | NM_007742 | 5’- CCCTGCCTGCTTCGTGTAAA -3’ |
|  |  | 5’- TCGTCTGTTTCCAGGGTTGG -3’ |
| *Acta2* | NM_007392 | 5’- ATCGTCCACCGCAAATGCTT -3’ |
|  |  | 5’- AACTGGAGGCGCTGATCCAC -3’ |
| *Pdgfrß* | NM_001146268 | 5’- CAGGACCTCTGGCTGAAGCA -3’ |
|  |  | 5’- TCTGGGAGGCAGAAGGGAGAT -3’ |
| *Postn* | NM_015784 | 5’- TCAAGGGCCTAGAAGACGATCA -3’ |
|  |  | 5’- AAACTCTGTGGTCTGGCCTCTG -3’ |
| *Thy1* | NM_009382 | 5’- CTTTCCCTCTCCCTCCTCCAAG -3’ |
|  |  | 5’- CGAGGGCTCCTGTTTCTCCTT -3’ |
| *Pla2g10* | *NM_008471* | 5’- CTTCTGGTCAGTCCTACACCTGTCA -3’ |
|  |  | 5’- GGCCACCAAGGCCACAATA -3’ |
| *Cdx1* | NM_009880 | 5’- GCCCGTCAAGGAGGAGTTTC -3’ |
|  |  | 5’- GGGCTGCAACTCAGAACAGG -3’ |
| *Cxcr4* | NM_009911 | 5’- CGGTAACCACCACGGCTGTA -3’ |
|  |  | 5’- TCTCCAGAACCCACTTCTTCAGAG -3’ |
| *Sox17* | NM_011441 | 5’- AGCCATTTCCTCCGTGGTGT -3’ |
|  |  | 5’- ACTGCTTCTGGCCCTCAGGT -3’ |
| *Gsc* | NM_010351 | 5’- CGCCTGGGCTACAACAGCTA -3’ |
|  |  | 5’- CCGGAGACACCAGTACAGAACC -3’ |
| *Cer1* | NM_009887 | 5’- ACTGTGCCCTTCAACCAGACCA -3’ |
|  |  | 5’- GGTGAATTTGGTGGGCGAGCA -3’ |
| *Epcam* | NM_008532 | 5’- GGTGGTGTCATTAGCAGTCATCG -3’ |
|  |  | 5’- TGTGGATCTCACCCATCTCCTT -3’ |
| *Ggt* | U30509 | 5’- CAGCTGCCTCAGACTCCAGAA -3’ |
|  |  | 5’- TTCCCATTCTCGTCCCTTGG -3’ |
| *Lgr5* | NM_010195 | 5’- GCAGACTACGCCTTTGGAAACC -3’ |
|  |  | 5’- GCTGTGGAGTCCATCAAAGCA -3’ |
| *Dlk1* | NM_010052 | 5’- GGATTCTGCGAGGCTGACAA -3’ |
|  |  | 5’- GCAGATGCACTGCCATGGTT -3’ |
| *Ins1* | NM_008386 | 5’- CAGACCTTGGCGTTGGAGGT -3’ |
|  |  | 5’- TCGAGGTGGGCCTTAGTTGC -3’ |
| *Ins2* | NM_001185083 | 5’- GCAGAAGCGTGGCATTGTAGA -3’ |
|  |  | 5’- TTATTCATTGCAGAGGGGTAGGC -3’ |
| *Amy2a5* | NM_001160151 | 5’- TGAAGGCTTTGTCAGCCACTTT -3’ |
|  |  | 5’- GTGCAATTGCCATCGACCTT -3’ |
| *Prss1* | NM_053243 | 5’- CACCAAGGTCTGCAACTATGTGG -3’ |
|  |  | 5’- ACAGTGACTGCAGAGGGATTGG -3’ |
| *Cela2a* | NM_007919 | 5’- CATCCGTCTTCACCAGGGTCT -3’ |
|  |  | 5’- GGGACAGTGGCAGTAATGTCTTCA -3’ |
| *Gapdh* | NM_008084 | 5’- CCAATGTGTCCGTCGTGGAT -3’ |
|  |  | 5’- TGCCTGCTTCACCACCTTCT -3’ |
| *Cyp1a1* | NM_009992 | 5’- CCTTCCGGCATTCATCCTTC -3’ |
|  |  | 5’- TTTCAGGCCGGAACTCGTTT -3’ |
| *Cyp1a2* | NM_009993 | 5’- AGGAGCTGGACACGGTGGTT -3’ |
|  |  | 5’- AGGTGTCCCTCGTTGTGCTG -3’ |
| *Cyp2a5* | NM_007812 | 5’- GCACTTCCTAGATGACAAGGGACA -3’ |
|  |  | 5’- CAGGCTCAACGGGACAAGAA -3’ |
| *Cyp2d22* | NM_001163472 | 5’- CCTCTCCTCGGCTGAGTTTCA -3’ |
|  |  | 5’- CGCCAGTGCATCAGGTTCA -3’ |
| *Cyp3a13* | NM_007819 | 5’- TCCTGCAGAACTTCACTGTCCA -3’ |
|  |  | 5’- TGGTTTCTGGTCCACAGGATACA -3’ |
| *Fbxo15* | NM_015798 | 5’- ATGGCCACGTGGAGAGAGG -3’ |
|  |  | 5’- TGCTGTGACACTGAACTCCCTTC -3’ |
| *viral-Gata4* |  | 5’- TATGGGCACAGCAGCTCCAT -3’ |
|  |  | 5’- GCAGAATTCGCCCTTTACGC -3’ |
| *viral-Hnf1a* |  | 5’- GTGGTACCTCACCCTTACCGAGTC -3’ |
|  |  | 5’- TGCAGCTGGCTCAGCTTAGAA -3’ |
| *vrial-Foxa3* |  | 5’- GTGGTACCTCACCCTTACCGAGTC -3’ |
|  |  | 5’- TGGTGGGCACAGGATTCACT -3’ |
| *episomal-Gata4* | | 5’- CAAGCAGGACTCTTGGAACAGC -3’ |
|  |  | 5’- TGAAAGCCATACGGGAAGCA -3’ |
| *episomal-Hnf1a* | | 5’- GATGGCCTCCTCTTCCCAGA -3’ |
|  |  | 5’- AGGCATTAAAGCAGCGTATCCA -3’ |
| *episomal-Foxa3* | | 5’- CAGAGCCTCTATTCCCGCTCTC -3’ |
|  |  | 5’- TGAAAGCCATACGGGAAGCA -3’ |
| *OriP/EBNA-1* | | 5’- CATCATCATCCGGGTCTCCA -3’ |
|  |  | 5’- CAGTGCTTGGGCCTTCTCCT -3’ |
